# Prothrombin complex concentrate for oral factor Xa inhibitor-associated intracerebral hemorrhage

**DOI:** 10.1016/j.rpth.2026.103361

**Published:** 2026-01-19

**Authors:** Mohammad Shamiea, Hassan Sbehi, Nadim Abu Rashed, Awan Kashua, Feda Fanadka, Eilam Rabina, Alex Osnis, Gilad Itchaki, Orly Avnery, Osnat Jarchowsky-Dolberg, Martin Ellis

**Affiliations:** 1 Department of Medicine A, Meir Medical Center, Tel Aviv, Israel; 2 Department of Neurology, Meir Medical Center, Tel Aviv, Israel; 3 Department of Neuroradiology, Meir Medical Center, Tel Aviv, Israel; 4 Blood Bank, Meir Medical Center, Tel Aviv, Israel; 5 Hematology Institute, Meir Medical Center, Tel Aviv, Israel; 6 Gray Faculty of Medical and Health Sciences, Tel Aviv University, Tel Aviv, Israel

**Keywords:** anticoagulants, factor Xa inhibitors, hemostasis, intracerebral hemorrhage, prothrombin complex concentrate

## Abstract

**Background:**

Factor Xa inhibitor-associated intracerebral hemorrhage (ICH) requires rapid anticoagulation reversal. Although andexanet alfa, a specific FXaI antidote, demonstrated efficacy in andexenet alfa for acute intracerebral hemorrhage (ANNEXA-I) trial, it was associated with a high thromboembolic rate. Consequently, 4-factor prothrombin complex concentrate (4F-PCC) is widely used, though real-world data remain limited.

**Objective:**

To assess the hemostatic effectiveness and safety of 4F-PCC for reversal of oral factor Xa inhibitors in patients with acute intracerebral hemorrhage.

**Methods:**

We conducted a single-center, retrospective observational study of consecutive patients with FXaI-associated ICH treated with 4F-PCC between January 2017 and May 2025. The primary endpoint was hemostatic efficacy according to ANNEXA-I criteria: hematoma expansion < 35%, National Institutes of Health Stroke Scale (NIHSS) score increase of <7 points, and absence of rescue therapy within 12 hours. The secondary endpoint was a stable neurological status (no worsening of the NIHSS score) at 48 hours. Safety outcomes included 30-day thromboembolic events and mortality.

**Results:**

Fifty-two patients (median age, 81 years; IQR, 75-87; 61.5% male) were included. Apixaban was the most frequent FXaI (86.6%), with atrial fibrillation as the main indication (94.3%). The median baseline hematoma volume was 5.45 mL (IQR, 2-21), and the NIHSS score was 4.5 (IQR, 1-6). The primary endpoint was achieved in 39 patients (75.0%; 95% CI, 61.1%-86.0%). Stable neurological status at 48 hours occurred in 37 patients (71.2%; 95% CI, 56.9%-82.9%). One thromboembolic event (deep vein thrombosis) occurred (1.9%; 95% CI, 0.0%-10.3%), and 12 patients (23.1%; 95% CI, 12.5%-36.8%) died within 30 days.

**Conclusion:**

4F-PCC achieved high hemostatic efficacy and low thromboembolic risk in FXaI-associated ICH. Mortality was comparable to ANNEXA-I, but thrombotic events were markedly lower, supporting current guideline recommendations for 4F-PCC use in this setting.

## Essentials

• Oral FXa inhibitors are widely used and may cause intracerebral bleeding.
• We conducted a single-center observational study using 4-factor prothrombin complex concentrate.
• Hemostatic outcomes were observed in a majority of patients with low thrombotic complications.
• This study provides real-world data on 4-factor prothrombin complex concentrate.

## 1 Introduction

Direct oral anticoagulants (DOACs), particularly factor (F)Xa inhibitors (FXaIs), are now the most frequently prescribed agents for atrial fibrillation and venous thromboembolism [[1], [2], [3], [4]]. Despite their favorable profile, DOACs carry significant bleeding risks, especially intracerebral hemorrhage (ICH). This potentially devastating complication [[5], [6], [7]] occurs in approximately 0.1% to 0.2% of DOAC-treated patients annually, with mortality rates of 15% to 25% [8,9].

Rapid anticoagulation reversal may limit hematoma expansion and improve neurologic outcomes. While dabigatran has an approved specific reversal agent (idarucizumab) [10], FXaIs present unique management challenges in the face of life- or organ-threatening bleeding. Andexanet alfa, the first specific FXaI antidote, demonstrated efficacy in the andexanet alfa for acute intracerebral hemorrhage ANNEXA-I trial (andexanet alfa for acute intracerebral hemorrhage) for ICH reversal; however, this came at the expense of arterial and venous thrombotic complications, which occurred in approximately 10% of patients [11]. These safety concerns, combined with subsequent real-world data showing limited availability and substantial costs, have prevented widespread clinical adoption and led to increased reliance on 4-factor prothrombin complex concentrate (4F-PCC) as an off-label alternative for FXaI reversal [[12], [13], [14], [15], [16]]. Despite recommendations for this indication, robust real-world data on the effectiveness and safety of 4F-PCC in this context remain limited.

In this retrospective study, we evaluate the hemostatic and clinical efficacy, as well as the safety, of 4F-PCC in FXaI-associated ICH using methodology consistent with the ANNEXA-I trial for andexanet alfa to provide standardized real-world evidence for clinical decision-making.

## 2 Methods

### 2.1 Study design and setting

We conducted a single-center, retrospective cohort study at the Meir Medical Center evaluating consecutive adult patients who received 4F-PCC (Octaplex, Octapharma) for reversal of oral FXaI-associated ICH from January 1, 2017, to May 31, 2025.

### 2.2 Patient selection

Inclusion criteria were: age ≥18 years, current FXaI therapy (apixaban, rivaroxaban, or edoxaban), neuroimaging-confirmed ICH, ingestion of a FXaI within 24 hours prior to presentation in the case of normal renal function or within 48 hours if the estimated glomerular filtration rate was <30 mL/min/1.73 m^2^, hematoma volume of 0.5 to 60 mL, National Institutes of Health Stroke Scale (NIHSS) score < 35 at presentation, and symptom onset ≤12 hours before presentation. Patients were required to fulfill all inclusion criteria for study eligibility.

Exclusion criteria were a Glasgow Coma Scale score < 7, concomitant antiplatelet therapy, or venous thromboembolism within 2 weeks of admission. Patients were excluded if any exclusion criterion was present.

### 2.3 Treatment protocol

Patients were managed according to a standardized institutional protocol. 4F-PCC was administered after approval by a consultant hematologist, at dose of 25 to 50 IU/kg ,intravenously over 15 to 20 minutes, reconstituted per the manufacturer’s instructions.

### 2.4 Data collection

Demographics information, comorbidities, indication for anticoagulation, the drug administered, time of last dose, time of onset of symptoms, neuro-imaging findings, administered dose of 4F-PCC, and 30-day bleeding or thrombotic events were extracted from electronic medical records using a standardized case report form.

### 2.5 Imaging assessment

All computed tomography scans were performed on Philips systems. Hematoma volume was measured independently by a neuroradiologist (F.F.) and a radiologist (N.A.) using the live-wire segmentation tool in the Philips PACS system. This semiautomated method allows precise contouring of hemorrhagic boundaries using local gradient information, improving accuracy over manual tracing. Hematoma expansion was expressed as the percentage change between baseline and the 12-hour follow-up scans.

### 2.6 Neurological assessment

A neurologist (H.S.) determined NIHSS scores at admission, 12 hours, and 48 hours.

### 2.7 Outcome measures

The primary endpoint was hemostatic efficacy using the definitions applied in the ANNEXA-I study: hematoma expansion < 35% at 12 hours (<20%: excellent; 20%-35%: good), an NIHSS score increase of <7 points within 12 hours, and no rescue therapy within 12 hours of admission . The secondary endpoint was a stable neurological status at 48 hours (no worsening of the NIHSS score from the 12-hour assessment). Safety endpoints were arterial or venous thromboembolism within 30 days and all-cause 30-day mortality.

### 2.8 Statistical analysis

Categorical variables were summarized as counts and percentages, and continuous variables as medians and IQRs. No imputation was performed for missing data. Because this was an observational study with a relatively small cohort, analyses were descriptive, and no formal hypothesis testing was undertaken.

For binary outcomes and proportions (eg, hemostatic efficacy, mortality, and thromboembolic events), exact binomial 95% CIs (Clopper–Pearson method) were calculated. For rare events and small denominators, 95% CIs are presented as exact values without continuity correction.

All analyses were performed using IBM SPSS Statistics version 29.0 (IBM Corporation).

### 2.9 Ethics

This study was approved by the Meir Medical Center Helsinki Committee. Patient-informed consent was waived due to the retrospective study design.

## 3 Results

From January 2017 to May 2025, 60 patients treated with 4F-PCC for FXaI-associated ICH were screened. Eight patients were excluded: 1 lacked follow-up imaging, 2 had Glasgow Coma Scale scores < 7, and 5 had hematoma volumes > 60 mL. The final analysis included 52 patients (Figure).

**Figure:**
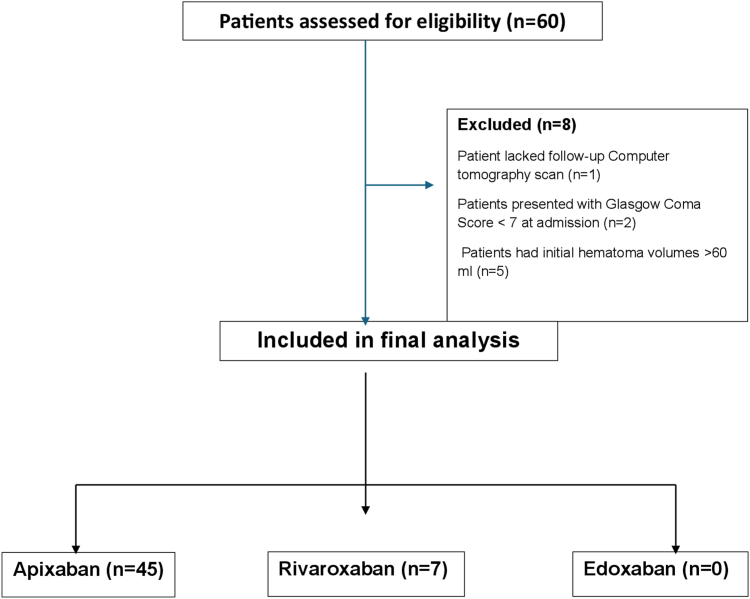
Study flow diagram. Flowchart illustrating patient screening, exclusion criteria, and the final cohort included in the analysis. Of the 60 patients assessed for eligibility, 8 were excluded due to a lack of follow-up computed tomography scan, a Glasgow Coma Scale score < 7 at admission, or an initial hematoma volume > 60 mL. The final study population included patients treated with apixaban (*n* = 45) and rivaroxaban (*n* = 7); no patients received edoxaban.

The median age was 81 years (IQR, 75-87), and 32 patients (61.5%) were men. All had received their last FXaI dose within 24 hours of admission. Apixaban was used in 45 patients (86.6%), and rivaroxaban in 7 (13.4%); none received edoxaban. Atrial fibrillation was the indication for anticoagulation in 49 patients (94.3%), and venous thromboembolism in 3 (5.7%). The median baseline hematoma volume was 5.45 mL (IQR, 2-21), the median NIHSS score was 4.5 (IQR, 1-6), and the median estimated glomerular filtration rate was 45 mL/min/1.73 m^2^ (IQR, 20-65). All patients received a 4F-PCC dose of 2000 IU (Table 1).

**Table 1:** Baseline characteristics and treatment details.

Characteristic	Value (*N* = 52)
**Demographics**	
Age, y	81 (75-87)
Male sex	32 (61.5)
**Anticoagulation details**	
Time from last FXaI dose to admission, h	≤24 for all patients
Type of FXaI	
Apixaban	45 (86.6)
Rivaroxaban	7 (13.4)
Edoxaban	0 (0)
Indication for anticoagulation	
Atrial fibrillation	49 (94.3)
Venous thromboembolism	3 (5.7)
**Baseline clinical characteristics**	
Hematoma volume, mL	5.45 (2-21)
NIHSS score	4.5 (1-6)
eGFR, mL/min/1.73 m^2^	45 (20-65)
**4F-PCC treatment details**	
4F-PCC dose, IU	2000 (2000-2000)
1500	2 (3.8)
2000	47 (90.4)
3000	3 (5.8)
Time from diagnosis to PCC administration, min	105 (60-140)
Time to follow-up CT scan, h	11.5 (10.5-13)

Data are presented as median (IQR) for continuous variables and *n* (%) for categorical variables.

4F-PCC, 4-factor prothrombin complex concentrate; CT, computed tomography; eGFR, estimated glomerular filtration rate; FXaI, factor Xa inhibitor; IU, International Units; NIHSS, National Institutes of Health Stroke Scale; PCC, prothrombin complex concentrate.

Supratentorial hemorrhage occurred in 47 patients (90.4%), most frequently in the frontal (16/52; 30.8%), temporal (9/52; 17.3%), and parietal (8/52; 15.4%) regions. Hemorrhage was infratentorial in 5 patients (9.6%; Table 2).

**Table 2:** Hematoma location (*N* = 52).

Location	*n* (%)
**Supratentorial**	47 (90.4)
Frontal	16 (30.8)
Temporal	9 (17.3)
Parietal	8 (15.4)
Basal nuclei	7 (13.5)
Occipital	3 (5.8)
Multilobar	3 (5.8)
Intraventricular	1 (1.9)
**Infratentorial**	5 (9.6)
Cerebellar	3 (5.8)
Brainstem	2 (3.8)

Supratentorial refers to brain structures above the tentorium cerebelli; infratentorial refers to structures below the tentorium cerebelli (cerebellum and brainstem).

The primary composite hemostatic efficacy endpoint—hematoma expansion < 35% at 12 hours, an NIHSS score increase of <7 points at 12 hours, and no rescue therapy ≤12 hours—was met in 39/52 patients (75.0%; 95% CI, 61.1%-86.0%). Among these, excellent efficacy (<20% expansion) was observed in 35 patients (67.3%; 95% CI, 52.9%-79.7%) and good efficacy (20%-35% expansion) in 4 patients (7.7%; 95% CI, 2.1%-18.5%).

Regarding the individual components of the composite outcome, hematoma expansion < 35% was achieved in 39/52 patients (75.0%; 95% CI, 61.10%-86.0%), an NIHSS score increase of <7 points occurred in 39/52 patients (75.0%; 95% CI, 61.1%-86.0%), and no rescue therapy was given in 50/52 patients (96.2%; 95% CI, 86.9%-99.5%).

The secondary endpoint, stable neurological status at 48 hours (no worsening of the NIHSS score), was achieved in 37/52 patients (71.2%; 95% CI, 56.9%-82.9%; Table 3).

**Table 3:** Primary efficacy outcomes (*N* = 52).

Outcome measure	*n* (%)
**Primary composite outcome**	39 (75.0)
**Individual components**	
Hematoma expansion < 35%	39 (75.0)
NIHSS score increase of <7 points at 12 h	39 (75.0)
No rescue therapy within 12 h	50 (96.1)
**Hemostatic efficacy classification**	
Excellent (<20% expansion)	35 (67.3)
Good (20%-35% expansion)	4 (7.7)
Poor (≥35% expansion)	13 (25.0)
**Secondary outcome**	
Stable neurological status at 48 h	37 (71.2)

NIHSS, National Institutes of Health Stroke Scale.

^a^Stable neurological status is defined as no decline in the NIHSS score.

### 3.1 Safety outcomes

A thromboembolic event occurred in 1/52 patients (1.9%; 95% CI, 0.0%-10.3%) and was a popliteal deep vein thrombosis on day 22. No arterial thromboembolic events were observed. Thirty-day all-cause mortality occurred in 12/52 patients (23.1%; 95% CI, 12.5%-36.8%; Table 4).

**Table 4:** Safety outcomes (*N* = 52).

Outcome	*n* (%)	Details
**Thromboembolic events**	1 (1.9)	-
Deep vein thrombosis	1 (1.9)	Day 22 post-PCC
Arterial thromboembolism	0 (0)	-
**30-d mortality**	12 (23.07)	

PCC, prothrombin complex concentrate.

## 4 Discussion

Our study demonstrates that 4F-PCC achieves effective hemostatic control in most patients who require reversal of the anticoagulation effect of FXaIs when ICH occurs in patients treated with these agents. The 75% hemostatic efficacy rate, with 67.3% of patients achieving excellent hemostatic control (hematoma expansion < 20%), suggests that 4F-PCC is a clinically effective intervention for this indication. Additionally, stable neurological status at 48 hours was observed in 71.2% of patients, while thrombotic complications were infrequent, occurring in 1.9% of patients, with no arterial thrombotic events observed.

These findings support the current guideline recommendations. The 2024 American College of Cardiology/American Heart Association/American College of Chest Physicians/Heart Rhythm Society guidelines on atrial fibrillation management acknowledge that while andexanet alfa is FDA-approved for apixaban- and rivaroxaban-associated life-threatening bleeding, 4F-PCC serves as a reasonable alternative at a dose of 25 to 50 U/kg for the reversal of the anticoagulant effect of oral FXaIs when andexanet alfa is unavailable [1]. Similarly, the 2024 International Society on Thrombosis and Haemostasis Scientific and Standardization Committee guidance endorses 4F-PCC at doses of 25 to 50 IU/kg when andexanet alfa is unavailable for rapid intervention in major bleeding scenarios [17].

The high cost of andexanet alfa in the United States, approximately $22,000 to $50,000 per treatment, and its limited availability have significantly restricted clinical use [18], leading to the uptake of 4F-PCC, which costs approximately $5670 per treatment. Recent comparative studies support this approach. Panos et al. [13] conducted a large, multicenter analysis of 1096 ICH patients, comparing andexanet alfa with 4F-PCC across 42 United States stroke centers. While andexanet alfa demonstrated higher hemostatic efficacy (87.8% vs 81.8%), it was associated with significantly higher thrombotic complications (7.9% vs 4.2%) [13].

Hemostatic efficacy was achieved in 75% of patients in our cohort, which was higher than the 67% rate reported in the landmark ANNEXA-I trial with andexanet alfa [11]. Furthermore, thrombotic complications occurred in only 1.9% of our patients compared with 10.3% in ANNEXA-I. In our study, the single event was a deep vein thrombosis occurring 22 days posttreatment, making a causal relationship with 4F-PCC uncertain. No arterial thrombotic events occurred in our series, in contrast to the significant arterial thrombotic burden observed with andexanet alfa.

The 30-day mortality rate (23.1%) in our cohort was comparable to that reported in ANNEXA-I (27.8%), reflecting the inherent severity of anticoagulant-associated ICH. Detailed mortality analysis (Supplementary Table S1) revealed that 58.3% of deaths were directly attributable to ICH complications, including hematoma expansion, postoperative complications, or severe neurological decline despite hemostatic control. The remaining 41.7% resulted from non-ICH medical complications, primarily sepsis and malignancy-associated coagulopathy. Importantly, no fatal thrombotic events related to 4F-PCC administration occurred, suggesting that mortality was driven by the natural course of ICH and underlying patient comorbidities rather than treatment-related adverse effects.

Several limitations of our study warrant acknowledgment. The single-center, retrospective design limits causal inference and introduces potential selection bias. Sample size constraints may limit the detection of rare adverse events, and the lack of anti-FXa activity measurements prevents correlation between pharmacodynamic reversal and clinical outcomes.

However, our study has significant methodological strengths. Independent neurological assessment by a neurologist at multiple time points, namely at admission within the first 24 hours and after 48 hours, ensures accurate NIHSS scoring and comprehensive neurological evaluation. Independent radiological review of all computed tomography scans and follow-up imaging by 2 radiologists provides precise hematoma volume measurements and expansion rate calculations. Finally, the 30-day follow-up period allows for the assessment of both immediate and delayed complications.

## 5 Conclusion

This study provides real-world evidence that 4F-PCC achieves hemostatic efficacy in most patients (75%) with FXaI-associated ICH. The remarkably low thrombotic complication rate (1.9%) and the absence of arterial thrombotic events, combined with widespread institutional availability, strongly support current guideline recommendations endorsing 4F-PCC as acceptable off-label therapy for FXa inhibitor reversal. These findings provide important data to guide clinical decision-making in emergency anticoagulation reversal, particularly in healthcare systems where balancing efficacy, safety, and cost-effectiveness is important. Given the widespread use of FXaIs and the critical nature of ICH, 4F-PCC represents a viable therapeutic option that can be effectively implemented in routine clinical practice.
