# Supplementary material for: Prothrombin complex concentrate for oral factor Xa inhibitor-associated intracerebral hemorrhage
Source: Res Pract Thromb Haemost. 2026 Jan 19;10(1):103361. doi: 10.1016/j.rpth.2026.103361 (PMC12906014; doi:10.1016/j.rpth.2026.103361)
Supplement: Supplementary Table S1 [file mmc1.docx]

**Supplementary materials**

**Detailed 30-day Mortality**

| **Patient** | **Age/Sex** | **Anticoagulant** | **Indication** | **Admission Date** | **Hematoma Volume (mL)**  **Baseline → 6-12h** | **NIHSS**  **Baseline → 48h** | **Date of Death** | **Clinical Course and Cause of Death** |
| --- | --- | --- | --- | --- | --- | --- | --- | --- |
| 1 | 78/M | Apixaban | AF | 12/05/25 | 0.9 → 0.9 | 5 → 8 | 20/05/25 | Initially presented with ischemic stroke that subsequently underwent hemorrhagic transformation. Despite stable hematoma volume, experienced progressive neurological deterioration with worsening NIHSS. Family elected for palliative care, and patient died from continued neurological decline. |
| 2 | 89/M | Rivaroxaban | AF | 11/05/25 | 21.9 → 20.5 | 3 → 3 | 25/05/25 | Achieved excellent hemostatic control with stable hematoma size and neurological status. Successfully discharged on 17/05/25 but readmitted several days later with severe sepsis secondary to pneumonia, which was the cause of death. |
| 3 | 85/F | Apixaban | AF | 18/06/24 | 6.8 → 6.9 | 6 → 15 | 22/06/24 | Demonstrated good hemostatic control initially but developed disseminated intravascular coagulation (DIC). Comprehensive imaging revealed multiple suspicious lesions in the liver consistent with underlying malignancy. Died from complications of presumed oncological disease with associated coagulopathy. |
| 4 | 81/M | Apixaban | AF | 14/04/23 | 3.0 → 6.7 | 3 → 5 | 08/05/23 | Initially achieved clinical stability and was discharged. Readmitted a week later with severe neurological deterioration. Neuroimaging revealed findings suggestive of brain metastases with associated hemorrhage. Transitioned to palliative care and died from complications of metastatic disease. |
| 5 | 88/M | Apixaban | AF | 14/04/23 | 4.6 → 60.0 | 6 → 42 | 16/04/23 | Experienced catastrophic hematoma expansion from 4.6 mL to 60 mL within 6 hours, accompanied by severe neurological deterioration with NIHSS increasing from 6 to 42. Died from complications of massive intracranial hemorrhage. |
| 6 | 88/F | Apixaban | AF | 12/03/23 | 5.0 → 5.1 | 12 → 42 | 07/04/23 | Despite stable hematoma size experienced severe neurological decline with NIHSS deteriorating from 12 to 42 and Glasgow Coma Scale decreasing to < 7. Required intubation for airway protection and died from progressive neurological failure. |
| 7 | 96/F | Apixaban | AF | 03/11/22 | 0.5 → 0.5 | 5 → 42 | 05/11/22 | Hematoma volume initially stable but suffered severe neurological deterioration with NIHSS increasing dramatically from 5 to 42. Glasgow Coma Scale decreased to < 7, with worsening of hemorrhage in follow up CT after 24 hours and patient died within 48 hours from neurological complications despite hemostatic control. |
| 8 | 83/F | Apixaban | AF | 05/11/23 | 12.3 → 170.0 | 18 → 45 | 06/11/22 | Suffered massive hematoma expansion from 12.3 mL to 170 mL. Neurological status deteriorated from NIHSS 18 to 45 with Glasgow Coma Scale < 7. Died after 24 hours from complications of catastrophic intracranial hemorrhage. |
| 9 | 88/F | Apixaban | AF | 29/04/22 | 36.0 → 46.0 | 1 → 10 | 08/05/22 | Presented with large initial hematoma that continued to expand. Patient and family declined all invasive interventions including surgical management. Experienced continuous neurological decline and died from progressive hemorrhage complications. |
| 10 | 77/M | Rivaroxaban | AF | 08/01/21 | 3.0 → 2.0 | 0 → 0 | 29/01/21 | Achieved excellent hemostatic control with slight hematoma reduction and stable neurological status (NIHSS 0). Successfully discharged but readmitted several days later with severe sepsis and died from infectious complications related to an underlying lymphoma. |
| 11 | 71/M | Apixaban | AF | 20/03/19 | 46.0 → N/A | 15 → N/A | 04/04/19 | Presented with large hematoma requiring surgical evacuation. Transferred to neurosurgical service for hematoma evacuation but failed to recover neurologically post-operatively. Remained intubated and died from post-surgical complications and failure to rehabilitate. |
| 12 | 68/M | Apixaban | DVT/PE | 9/12/22 | 1.4 → 1.7 | 0 → 35 | 26/12/22 | Presented with ICH to hospital and developed DIC two days later, then continued to deteriorate with GCS < 7 and died from complications of underlying hepatic carcinoma and coagulopathy. |

*Abbreviations: AF, atrial fibrillation; CT, computed tomography; DIC, disseminated intravascular coagulation; DVT/PE, deep vein thrombosis/pulmonary embolism; F, female; GCS, Glasgow Coma Scale; ICH, intracranial hemorrhage; M, male; mL, milliliters; N/A, not*

**Comparison Between Present Study and ANNEXA-I Trial**

| **Characteristic** | **Present Study**  **(4F-PCC)** | **ANNEXA-I**  **(Andexanet alfa)** |
| --- | --- | --- |
| Age, years | 81 | 78.9 |
| Male sex, % | 61.5 | 58 |
| Atrial fibrillation, % | 94.3 | 90.2 |
| Hematoma volume at admission, mL | 5.45 | Not reported |
| NIHSS at admission | 4.5 | Not reported |
| Primary hemostatic efficacy, % | 75 | 67 |
| Thromboembolic events, % | 1.9  *(No arterial events)* | 10.3 |

*Abbreviations: 4F-PCC, four-factor prothrombin complex concentrate; NIHSS, National Institutes of Health Stroke Scale*
